# Equivalence Class Formation in Adults with Severe Behavioral Problems

**DOI:** 10.1007/s40732-023-00540-6

**Published:** 2023-04-12

**Authors:** Jesús Alonso-Vega, María Xesús Froxán-Parga, Erik Arntzen

**Affiliations:** 1 Psychology Department, Faculty of Biomedical and Health Sciences, Universidad Europea de Madrid, Villaviciosa de Odon, Spain; 2 Present Address: Department of Biological and Health Psychology, Universidad Autónoma de Madrid, Madrid, Spain; 3 Department of Behavioral Science, Oslo Metropolitan University, Oslo, Norway

**Keywords:** Stimulus equivalence, Training structures, Training protocols, Severe mental disorders, Severe behavioral problems

## Abstract

Stimulus equivalence is a behavioral phenomenon that has been related to complex human behavior (e.g., remembering, cognitive functioning, and symbolic behavior). As a rule, people diagnosed with severe mental disorders (e.g., schizophrenia, bipolar disorder) that exhibit delusional and hallucinatory behavior, and disorganized speech have shown cognitive impairment (e.g., processing speed, reasoning/problem solving). Not enough research has analyzed the stimulus equivalence performance in this population. This study aims to investigate the stimulus equivalence performance in adults diagnosed with severe mental disorders. In particular, this study analyzes the many-to-one (MTO) and one-to-many (OTM) training structures effects, and the simultaneous (SIM) and the simple-to-complex (STC) training and testing protocol effects on equivalence class formation in this population. To achieve it, we analyzed the behavior of 18 participants diagnosed with severe mental disorders in three different conditions (Condition 1 OTM/SIM; 2 MTO/SIM; and 3 MTO/STC). Behavior consistent with stimulus equivalence was found in 11 out of 13 participants who had finished the study (5 participants decided to leave before completing the tasks). STC yielded better results than the SIM protocol. No differences were found between MTO and OTM training structures. Implications and suggestions for further research have been discussed.

Schizophrenia, bipolar disorder, borderline personality disorder, and some types of dementia are diagnostic labels under the umbrella of *severe mental disorders.* People diagnosed with this type of psychological problem usually exhibit delusional and hallucinatory behavior, disorganized speech, and cognitive impairment (e.g., verbal memory, processing speed, reasoning/problem solving, symbol coding; Bedi et al., 2015; Hill et al., 2013; Mota et al., 2012; Zaninotto et al., 2015). These behaviors typically affect multiple areas of adaptive functioning (e.g., daily life activities, self-care behaviors, job performance; Marcus & Cather, 2016; Strassnig & Harvey, 2019). Diagnostic labels are developed from a biomedical model of psychological problems that imply that these behaviors are symptoms of an underlying biological problem (Deacon & McKay, 2015). However, these biological theories of psychological problems failed to elucidate the biological basis of these diagnostic labels (Frances & Nardo, 2013), people diagnosed with the same problem engage in different behavioral problems (Deacon, 2013), and the diagnostic does not allow for planning a successful intervention (Wilder, 2009). A behavioral analytic account of *severe behavioral problems*^1^ related to these diagnostics does not imply a common underlying biological or mental problem, and is centered on analyzing environmental variables of which these behaviors are function. Also, this type of analysis suggests, among other characteristics (seeFerreira et al., 2021; Madden et al., 2016); an idiographic study of these problematic behaviors, a functional-analysis based intervention, and the use of experimental behavior analytic findings to understand and develop behavioral programs for these serious behavioral problems (Cautilli, 2018; Iwata & Dozier, 2008). Our study may contribute to this discussion by examining how experimental parameters affect complex behaviors in people with schizophrenia and other severe mental disorders.

Within behavior analysis, complex human behaviors such as remembering, cognitive functioning, and symbolic behaviors have been related to stimulus equivalence phenomenon (Palmer, 1991; Wilkinson & McIlvane, 2001) and have been studied by using conditional discrimination procedures (Brogård-Antonsen & Arntzen, 2020; Steingrimsdottir & Arntzen, 2014). *Stimulus equivalence* is a behavioral phenomenon defined by the emergence of untrained (i.e., novel) behaviors after a specific baseline training (Sidman, 2009; Sidman & Tailby, 1982). As a rule, conditional discrimination techniques are used during training to teach a set of interrelated baseline relations (e.g., AB, BC, and CD). After that baseline training, participants are tested to respond to untrained relations (e.g., A = A, known as *reflexivity*; B = A, *symmetry;* and A = D, *transitivity*). Stated in more precise language, a set of N stimuli arranged in N-1 baseline trained relations can produce the emergence of N^2^-N + 1 untrained relations (Fields et al., 2020). If an individual performs according to *reflexivity*, *symmetry,* and *transitivity* during the testing trials, it is said that they have formed classes of equivalent stimuli and, therefore, they have performed in accordance with stimulus equivalence (Sidman & Tailby, 1982).

This type of research paradigm has been useful to study complex human behavior performance. For example, stimulus equivalence has been analyzed in children diagnosed with autistic spectrum disorder and other developmental disabilities; adults without mental diagnostics; patients with dementia and Alzheimer’s; and elderly people (Arntzen, 2012). Data yielded by these experiments are useful for expanding the basic knowledge about conditions that allow individuals to show performance characterized as stimulus equivalence (e.g., participant characteristics, training structures, testing protocols) and thus for the effective arrangement of stimulus equivalence procedures in behavioral programs (e.g.,Bolanos et al., 2020; Giannakakos et al., 2021; Mullen et al., 2017; Steingrimsdottir & Arntzen, 2015). Adults diagnosed with *serious mental illness* can benefit from this experimental and applied behavior analysis. However, to our knowledge, only one study (González Pando et al., 2016) has analyzed the stimulus equivalence class formation in patients diagnosed with severe mental illness.

González Pando et al. (2016) conducted a preliminary experimental study to analyze the equivalence class performance in adults diagnosed with schizophrenia. In this experiment, they arranged a training for the emergence of two 3-member equivalence classes of abstract stimuli. They trained four baseline relations (A1B1, A2B2, B1C1, and B2c2) to eight inpatients from a public mental health hospital using matching to sample procedures. The training structure used was a linear series structure (e.g., training AB baseline relations first and BC baseline relations then), and they used a simple-to-complex protocol (STC), to test for the emergence of novel relations (e.g., Fienup et al., 2015). In the STC, training of AB relations is followed by the test for the symmetrical relations (BA); the second baseline relation (BC) is then trained, followed by the test for the emergence of symmetrical relations (CB), transitive relations (AC), and equivalence relations (CA). After the baseline training, half of the participants showed a performance according to stimulus equivalence during testing probes. This study represents one of the first attempts to experimentally study the equivalence class performance in adults diagnosed with schizophrenia; however, it has some experimental flaws. They used a mastery criterion of ≥ 70% correct responding during training and testing. This mastery criterion is not rigorous enough and it does not allow experimenters to confirm that the equivalence classes were formed. Another flaw of this experiment is the number of participants and the high rate of experimental withdrawal; they started the experiment with eight participants and just four of them finished the experimental tasks. These flaws severely limit the interpretation of the results. But, despite the flaws of this experimental study González Pando et al. have proved the capability of some members of this population to show the emergence of untrained relations and have opened the way for further experimental analysis and the possibility to add equivalence-based instructions to the clinician treatment tools of this population.

Moreover, the study of other derived relations with this clinical population is also scarce (Hendriks et al., 2020). There are pilot experiments dedicated to assessing the already established deictic relational responding in adults diagnosed with schizophrenia (Villatte et al., 2010) and adults who present psychosis-related problems (McEnteggart et al., 2017), and to study the differences between hearing-voices individuals and a control group using the implicit relational assessment procedure (IRAP; McKenna et al., 2007).

The study of stimulus equivalence and derived relations in people diagnosed with severe mental illness is a promising area of research that needs further investigation. Studying the performance of this population on stimulus matching tasks and analyzing the variables that could improve their performance could help us to improve behavioral interventions for these specific problems. In both basic and applied research on stimulus equivalence formation, an extensive literature is available regarding different procedural variables (e.g., training structures, training protocols, number of classes and/or members, simultaneous and delayed matching to sample) that could enhance the stimulus class formation (cf., Arntzen, 2012). For instance, three training structures have traditionally been used to train the baseline relations: linear series (LS; e.g., training AB and BC baseline relations), many-to-one (MTO; e.g., training BA and CA baseline relations), and one-to-many (OTM; e.g., training AB and AC baseline relations). Several empirical studies have been conducted to grasp how the training structures affect the results of equivalence (e.g.,Ayres-Pereira & Arntzen, 2021; Fields & Moss, 2007; Smeets & Barnes-Holmes, 2005). In general, the LS training structure is considered the least effective in establishing equivalence classes; although there is still an ongoing debate about the main differential effects of MTO and OTM to improve the performance of equivalence class formation (Arntzen, 2012). Another procedural variable that interacts with the effects of different training structures is the training protocol (i.e., how the training and testing trials are sequenced; Fields et al., 1997; Imam, 2006). Two of the most studied training protocols are the simultaneous protocol (SIM) and the simple-to-complex protocol (STC; e.g., Fienup et al., 2015). Both protocols have proved their efficacy in establishing equivalence classes among different populations. However, as previously stated, there is a lack of experimental evidence on stimulus equivalence in people diagnosed with severe mental illness. Therefore, experiments devoted to analyzing how these procedural variables affect performance in equivalence classes could expand this experimental literature and stimulate further research.

The general purpose of this study was to assess the equivalence class performance of adults diagnosed with severe behavioral problems and variables that could improve their performance. In particular, the main purpose of this article was to analyze the MTO and OTM effects and the SIM and STC effects on equivalence class formation in individuals diagnosed with severe mental illness. To achieve it, we analyzed equivalence class formation in this population (i.e., people diagnosed with severe mental disorders who present severe behavioral problems such as delusions, hallucinations, and impairments in cognitive functioning) by comparing (1) the effects of OTM and many-to-one (MTO) in equivalence performance and (2) comparing the effects of simultaneous training protocol (SIM) and simple-to-complex testing protocol (STC). This experiment would replicate the results and solve some experimental flaws of González Pando et al. (2016) study by using a stricter learning criterion and using three stimulus comparisons rather than two (Arntzen, 2012). Also, it would be useful to know more about the effect of different training structures, and training and testing protocols on the performance of people with severe behavioral problems.

## Method

### Participants

Eighteen adults diagnosed with severe mental disorders accepted to participate in this study. Once they were accepted, they were randomly assigned to one of the three experimental conditions. The age, sex, diagnostic label, and education level of each participant were registered (we have labeled as “high educational level” the participants who have received a university degree, “middle educational level” for high school graduate or other professional certifications, and “low educational level” for elementary school; see Table 1). All participants were recruited from the same public-funded work rehabilitation center (WRC) based in Spain; in this center, they receive psychological treatment, occupational therapy, and pharmacological treatment under the supervision of a mental health system. The first contact with participants was made by the center’s psychologist or clinical director. Once they agreed to participate, the first author explained and read with them the consent form. They were informed of their rights to withdraw at any time without penalty; none of them presented problems understanding the consent form's details, and no participant needed nor had a legally authorized representative or similar legal figures. None of the participants reported having experience in this type of experimental procedure. At the end of the experiment, the first author debriefed the experimental task with the participants. Two ethics committees (i.e., WRC and UAM’s ethics committees) have approved this experiment’s procedures. All current COVID-19 restrictions were followed.

**Table 1:** Participants’ Characteristics

Condition	ID	Sex	Age	Diagnostic Label	Education Level ^a^
1	1001	M	55	Bipolar Disorder	High
	1002	M	54	Schizophrenia and related psychosis	Middle
	1003	F	46	Schizophrenia and related psychosis	High
	1004	M	27	Schizophrenia and related psychosis	Middle
	1005	M	38	Personality Disorder	Low
	1006	F	43	Schizophrenia and related psychosis	Low
Total	*n* = 6		*SD* = 9.58	BD = 17%; SCH = 66%; PD = 17%	L = 33%; M = 33%; H = 33%
2	2001	F	55	Personality Disorder	Low
	2002	M	57	Bipolar Disorder	High
	2003	F	32	Bipolar Disorder	Low
	2004	M	32	Schizophrenia and related psychosis	Low
	2005	M	41	Bipolar Disorder	Middle
	2006	M	57	Bipolar Disorder	High
Total	*n* = 6	M	*SD* = 11.1	BD = 66%; SCH = 17%; PD = 17%	L = 50%; M = 17%; H = 33%
3	3001	M	50	Schizophrenia and related psychosis	Middle
	3002	F	37	Schizophrenia and related psychosis	Low
	3003	M	42	Personality Disorder	Middle
	3004	M	39	Schizophrenia and related psychosis	High
	3005	F	41	Schizophrenia and related psychosis	Low
	3006	F	28	Personality Disorder	Middle
Total	*n* = 6		*SD* = 6.55	BD = 0%; SCH = 66%; PD = 34%	L = 33%; M = 50%; H = 17%

*BD* bipolar disorder, *SCH* Schizophrenia and related psychosis, *PD* Personality Disorder, *L* Low (elementary school), *M* Middle (high school graduate), *H* high (university degree)

### Equipment and Setting

The experiment was carried out in a quiet and small room (5 m × 2.5 m) inside the work rehabilitation center. The room was equipped with a table (2 m x 1 m), two chairs, and a laptop in which the tasks were conducted. Also, according to the COVID-19 measures, a pump bottle dispenser of hand sanitizer gel was available for the participant at any time. The windows were partially open, and the keyboard was cleaned after and before each participant.

The stimuli, behavioral recording, and instructions were presented using *PsychoPy2,* an open-source software package (Peirce et al., 2019). The participants were asked to use the keyboard to advance during the experiment and select the stimuli during the MTS procedures. We scheduled one session per participant, each session lasted no more than 180 min, and participants were instructed to ask for a break whenever they wanted it. No participant asked for a break and sessions lasted no longer than 85 min, but some participants decided to withdraw (see Results section for further details).

### Stimuli

**Fig. 1:**
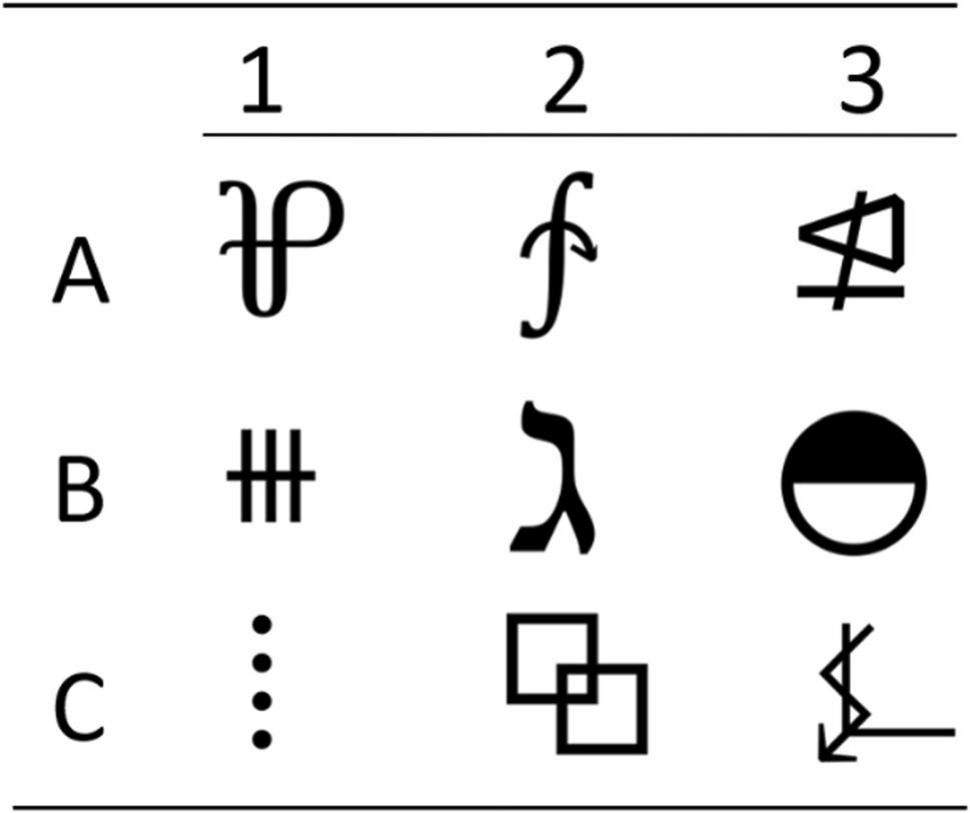
Stimulus Set. *Note.* Each stimulus was labeled with a number and a letter. Numbers indicate the class (e.g., class 1, class 2, and class 3) expected for each stimulus during the test for emergent relations, and letters indicate the exemplar of the class

Stimuli consisted of nine abstract shapes (see Fig. 1). These stimuli had no meaning for the Spanish verbal community of our participants. The classes of equivalent stimuli to form were randomly made before the experiment, so there was no preexperimental relation between the stimuli; nevertheless, it was measured during the pretest phase of the procedure.

### Design

We used a single-subject pretest/posttest design (Sidman, 1971) with three different conditions, each replicated with six participants. In all conditions, participants trained six baseline relations using a simultaneous matching-to-sample (SMTS) procedure and were tested for the emergence of six symmetry relations and six transitivity relations (i.e., we expected 12 relations to emerge to confirm the equivalence class formation). Moreover, we manipulated three procedural variables to produce each experimental condition (see Table 2). In Condition 1, participants trained the baseline relations BA and CA, using an MTO training structure, and were tested for the emergence of symmetry and equivalence relations (i.e., AB, AC, BC, and CB) using a SIM testing protocol. In Condition 2, participants trained the baseline relations AB and AC, using an OTM training structure, and were tested for the emergence of symmetry and equivalence (i.e., BA, CA, AB, CB) using a SIM testing protocol. Finally, in Condition 3, we used the MTO training structure with a simple-to-complex (STC) protocol. Conditions 1 and 2 served to compare training structures (MTO and OTM) effects under a SIM protocol. Conditions 2 and 3 served to compare testing protocols (SIM and STC) effects under an MTO training structure.

**Table 2:** Characteristics of the Experimental Conditions

Condition	Training structure	Training Protocol	MTS procedure	Training MC	Test MC
1	OTM	Simultaneous	SMTS	12 CC	16/18
2	MTO	Simultaneous	SMTS	12 CC	16/18
3	MTO	Simple-To-Complex	SMTS	12 CC	16/18

*OTM* One-To-Many, *MTO* Many-To-One, *SMTS* Simultaneous Matching to Sample, *MC* Mastery Criteria

### Procedure

#### Trial Structure

Each trial consisted of a matching-to-sample procedure in which an observing response to the sample was required. The sample stimuli appeared in the upper part of the screen. The participants were asked to press the up key of the keyboard when the sample was on the screen (i.e., observing response). This was followed by the presentation of three comparisons in the right, center, and left part of the screen. The participants selected these stimuli using the keyboard's right, low, and left keys. During the baseline training, a correct selection by the participant was followed by the presentation of the written words “Yes” and “Perfect” colored in green. If participants made a wrong selection during the training, the word “No” appeared on the screen colored in red. These words were in the center of the screen for 1.5 s and followed by a blank screen for 500 ms.

#### Pretest

The pretest evaluated if participants would respond according to equivalence before training. In this phase, first, participants read the following instruction printed on the screen (translated from Spanish): “When you see a figure at the top of the screen, please, press the up key. After three figures appear on the screen, please, select one of them with the correspondent right, low, and left keys.” Then, participants were exposed to a block of 54 trials without any differential consequences after each selection. During this phase of the procedure, all relations between stimuli were presented randomly. If one participant scored more than 18 correct trials (approximately 33% of correct responses), they would be removed from the experiment.

#### Baseline Training

In this phase, the following instruction was presented on the screen (translated from Spanish): “Now you have to learn how to respond correctly to the symbols. We are going to deliver you feedback after each choice.” This phase consisted in establishing the baseline relations on each condition (see Table 3). For example, during this phase, participants in Condition 1 were trained to establish six stimulus relations: AB relations (e.g., A1B1, A2B2, and A3B3) were established using matching-to-sample trials (i.e., A1/B1B2B3, A2/B1B2B3, and A3/B1B2B3; the line under the stimulus indicates experimenter-defined comparisons) and AC relations (i.e., A1C1, A2C2, and A3C3) were established using matching to sample trials (i.e., A1/C1C2C3, A2/C1C2C3, and C3/C1C2C3). The baseline training followed the same logic on all the training phases shown in Table 3. The OTM training structure was used in Condition 1; and MTO training structure in Conditions 2 and 3. The mastery criteria in this phase for all conditions was 12 consecutive correct responses.

**Table 3:** Experimental Phases and Stimulus Relations

Condition	Phases	Trials	Prob. of Consequences (%)	Trials per Block	Mastery Criteria
1	Training	AB	100	-	12 CC
	Training	AC	100	-	12 CC
	Mix. Testing	(BS) AB, AC; (SYM) BA, CA; (EQ) BC, CB	0	54	50 C
2	Training	BA	100	-	12 CC
	Training	CA	100	-	12 CC
	Mix. Testing	(BS) BA, CA; (SYM) AB, AC; (EQ) BC, CB	0	54	50 C
3	Training	BA	100	-	12 CC
	Testing	(SYM) AB	0	9	8 C
	Training	CA	100	-	12 CC
	Testing	(SYM) AC	0	9	8 C
	Mix. Training	BA, CA	100	-	12 CC
	Mix. Testing	(SYM) AB, AC	0	18	16 C
	Mix. Testing	(EQ) BC, CB	0	18	16 C
	Mix. Testing	(BS) BA, CA; (SYM) AB, AC; (EQ) BC, CB	0	54	50 C

*BS* Baseline, *SYM* Symmetry, *EQ* Equivalence, *CC* Consecutive Correct, *C* Correct responses. During the mixed training and testing phases, all trials were presented randomly

#### Baseline and Emergent Relations Tests

After mastering the baseline relations, participants were tested for the emergence of three 3-member equivalence classes. Before starting this phase, the following instruction was presented on the screen (translated from Spanish): “Now, please, choose accordingly to what you previously learned.” This phase consisted of testing (i.e., without consequences) emergent relations between stimuli.

For example, six trained baseline relations (i.e., A1B1, A2B2, A3B3, A1C1, A2C2, and A3C3); six untrained symmetry relations (i.e., B1A1, B2A2, B3A3, C1A1, C2A2, and C3A3); and 6 untrained equivalence relations (i.e., B1C1, B2C2, B3C3, C1B1, C2B2, and C3B3) were tested during the mixed testing of the Condition 1 (see Table 3). The simultaneous testing protocol was used with Conditions 1 and 2; a simple-to-complex (STC) testing protocol was used with the participants of Condition 3 (see Table 3). The mastery criteria for these tests were > 90% of correct responses. If a participant did not meet the mastery criteria in one of the test blocks, the task ran until all trials were presented (e.g., 54 trials during the mixed testing), and they were exposed to another training phase. Condition 3 participants that did not meet the mastery criteria during the mixed testing (i.e., (SYM) AB, AC) were exposed again to the mixed training phase (i.e., B1A1, B2A2, B3A3, C1A1, C2A2, C3A3). Also, if a participant did not meet the mastery criteria after three training–testing cycles, the experimental session ended.

### Response Measurement and Data Analysis

We registered the number of correct responses and incorrect responses (according to the stimulus classes presented in Fig. 1) during training phases; the number of trials to meet the required criteria per participant in each phase; and response speed (i.e., 1/reaction time; Whelan, 2008). Pretest and testing phase performances of each participant were categorized as “Passed” or “Failed” based on the mastery criteria required for each phase (see Table 3).

## Results

**Fig. 2:**
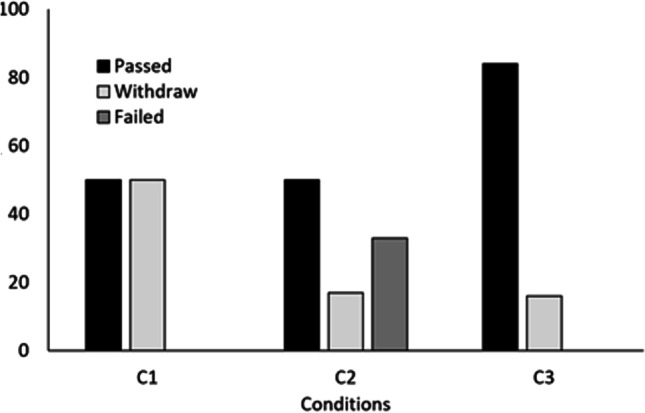
Participant’s General Performance per Condition. *Note.* Passed = participants who have met the mastery criteria required for the equivalence class formation; Failed = participants who did not form equivalence classes during the three experimental cycles; BD = bipolar disorder; SCH = Schizophrenia and related psychosis; PD = Personality Disorder

Overall, 61,11% of participants have formed equivalence classes (i.e., 11/18; Condition 1 = 3/6, Condition 2 = 3/6, and Condition 3 = 5/6); 27,7% of participants have withdrawn during the experiment (i.e., 5/18; Condition 1 = 3/6, Condition 2 = 1/6, and Condition 3 = 1/6); and 11% of participants have not formed equivalence classes (i.e., 2/18; Condition 1 = 0/6, Condition 2 = 2/6, and Condition 3 = 0/6; see Fig. 2). Moreover, no participant met the exclusion criterion in the pretest phase.

### Performance during Training Trials

**Fig. 3:**
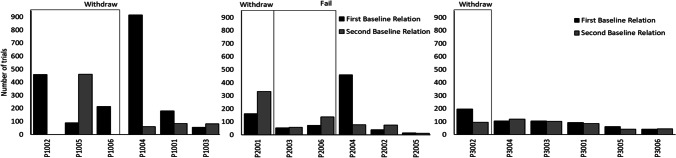
Trials to Learn Each Trained Baseline Relation. *Note.* This figure represents the number of trials required by each participant (Condition 1 left, Condition 2 center, and Condition 3 right plot)

Tables 4, 5, and 6 show each participant’s performance per condition. Table 4 displays the performance of Condition 1 (OTM-SIM) participants and the time that they spent during the experimental tasks. This condition generated more withdrawals than all other conditions, 50% compared to the 16,6% in the other conditions. On average, participants in this condition needed more time to finish the experiment (Condition 1 M = 57.8, *SD* = 13.2; Condition 2 M = 42.5, *SD* = 18.09; Condition 3 M = 34.6; *SD* = 10.27). Also, Condition 1 participants needed more trials to learn baseline relations, but that difference could be explained by the performance of P1004 (see Fig. 3).

**Table 4:** Condition 1 (OTM-SIM) Participant’s Number of Trials in Each Phase

P	Cycle 1					Cycle 2					Cycle 3					(min)
	AB	AC	BSL	SYM	EQ	AB	AC	BSL	SYM	EQ	AB	AC	BSL	SYM	EQ	
1001	166	70	5	9	13	15	14	**17**	**18**	**17**	-	-	-	-	-	56
1002		-	-	-	-	-	-	-	-	-	-	-	-	-	-	48
1003	41	43	15	14	7	14	39	**18**	**18**	**16**	-	-	-	-	-	52
1004	891	34	15	14	13	12	13	18	16	13	12	12	**18**	**18**	**16**	85
1005	89		-	-	-	-	-	-	-	-	-	-	-	-	-	61
1006		-	-	-	-	-	-	-	-	-	-	-	-	-	-	45

*AB* and *AC* baseline training, *BSL* baseline probes, *SYM* symmetry probes, *EQ* equivalence probes. Bold and underlined cells mean that the participant has met the mastery criteria during emergence relations tests. Strikethrough cells mean that the participant withdrew from the experiment. Finally, the rightmost column shows the total session duration

**Table 5:** Condition 2 (MTO-SIM) Participant’s Number of Trials on Each Phase

P	Cycle 1					Cycle 2					Cycle 3					(min)
	BA	CA	BSL	SYM	EQ	BA	CA	BSL	SYM	EQ	BA	CA	BSL	SYM	EQ	
2001	57	162	11	10	0	42	69	12	9	2	63		-	-	-	72
2002	12	45	10	12	3	12	15	11	10	5	15	15	**18**	**17**	**17**	38
2003	27	27	9	9	0	13	18	18	14	5	12	12	**18**	**17**	4	45
2004	303	34	13	11	6	37	27	13	11	8	19	16	**17**	**18**	**16**	55
2005	14	12	**18**	**18**	**18**	-	-	-	-	-	-	-	-	-	-	15
2006	22	32	8	6	10	17	69	9	10	8	34	35	9	8	9	30

*BA and CA* baseline training, *BSL* baseline probes, *SYM* symmetry probes, *EQ* equivalence probes. Bold and underlined cells mean that the participant has met the mastery criteria during emergence relations tests. Strikethrough cells mean that the participant withdrew from the experiment. Finally, the rightmost column shows the total session duration

**Table 6:** Condition 3 (MTO-STC) Participant’s Number of Trials on Each Phase

P	Cycle 1		Cycle 2		Cycle 1		Cycle 2		Cycle 1		Cycle 2		Cycle 3		C. 1		C .2		Cycle 1			
	T.BA	SYM AB	T.BA	SYM AB	T. CA	SYM AC	T. CA	SYM AC	BA CA	SYM AB,AC	BA CA	SYM AB,AC	BA CA	SYM AB,AC	EQ BC	EQ CB	EQ BC	EQ CB	BSL	SYM	EQ	min
3001	53	**9**	-	-	46	**9**	-	-	66	16	12	**17**	-	-	3	6	7	**8**	**18**	**18**	**16**	40
3002	113	7	19	**8**	31	**8**	-	-	90	14	15	12	21		6	4	-	-	-	-	-	43
3003	33	7	12	**9**	30	5	13	**9**	90	12	30	**16**	-	-	**9**	7	-	-	**18**	**18**	**18**	41
3004	56	5	39	**9**	109	**9**	-	-	20	**18**	-	-	-	-	**9**	**9**	-	-	**18**	**18**	**18**	43
3005	43	**9**	-	-	12	6	12	**9**	36	**18**	-	-	-	-	**9**	**9**	-	-	**18**	**18**	**18**	17
3006	29	**9**	-	-	32	**9**	-	-	12	**18**	12	**18**	-	-	2	3	7	**9**	**17**	**18**	**18**	24

*T.* trained relations, *BSL* baseline probes, *SYM* symmetry probes, *EQ* equivalence probes. Bold and underlined cells mean that the participant has met the mastery criteria during emergence relations tests. Strikethrough cells mean that the participant withdrew from the experiment. Finally, the rightmost column shows the total session duration

High variability can be found in Condition 1 (OTM-SIM) and Condition 2 (MTO-SIM) participants’ performance. In general, Condition 3 (MTO-STC) participants’ performance is more stable than in the other conditions (see Fig. 3). For example, Conditions 1 and 2 have shown more differences between participants and more differences between trials needed to learn the first and second baseline relations. On the other hand, Condition 3 participants have shown a similar number of trials to learn baseline relations, and they needed an equal number of trials to learn each baseline relation.

### Performance during Tests

**Fig. 4:**
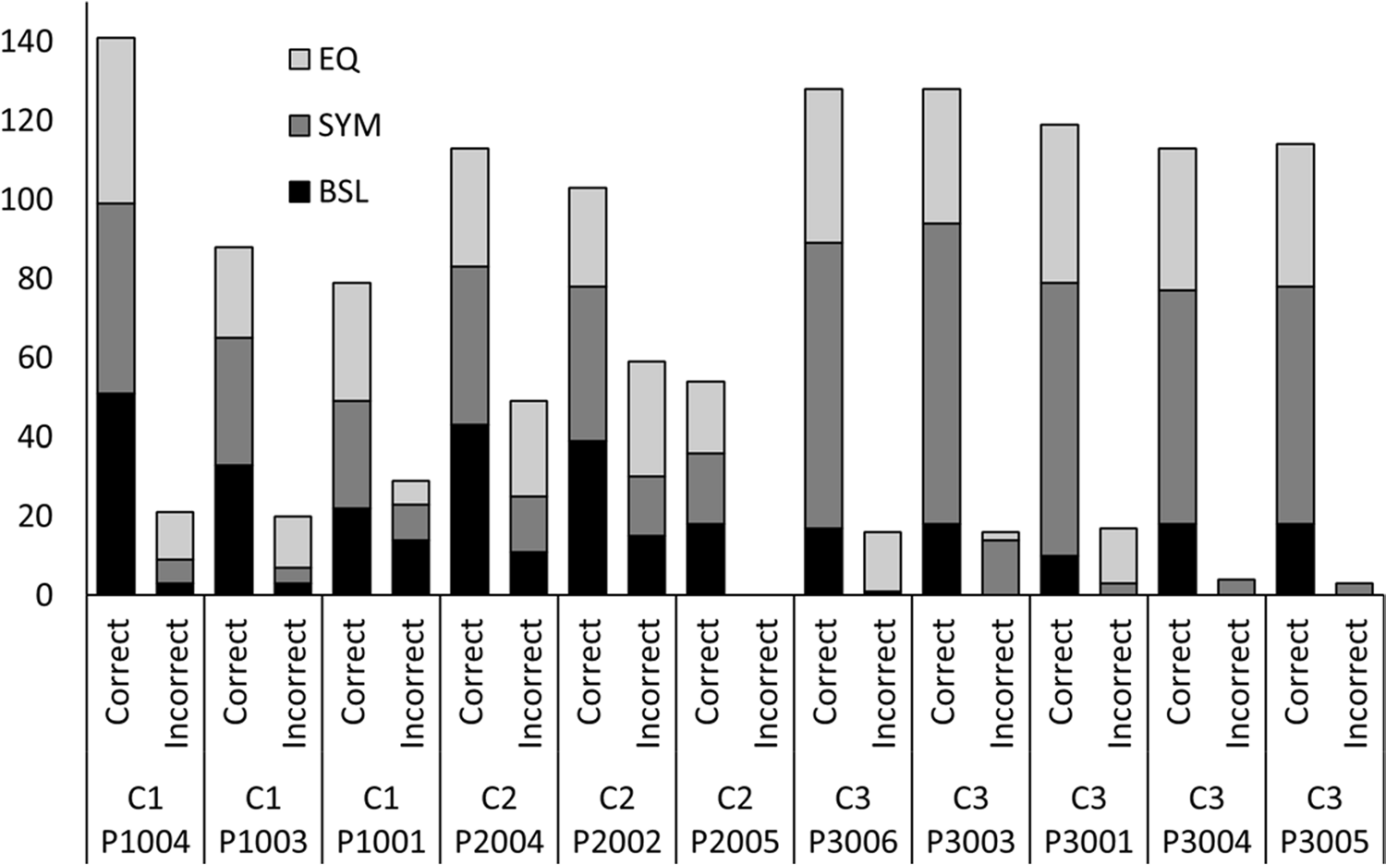
Number of Correct and Incorrect Trials per Participant during Emergence Probes. *Note.* This graph displays results obtained only by participants who have met the mastery criteria for equivalence class formation. EQ = equivalence probes; SYM = symmetry probes; BSL = baseline probes. C = condition; P = participant

In general, Condition 3 (MTO-STC) has had higher yields in emergence probes than the other experimental conditions (see Fig. 4). Moreover, Conditions 1 and 2 had more incorrect responses than Condition 3.

**Fig. 5:**
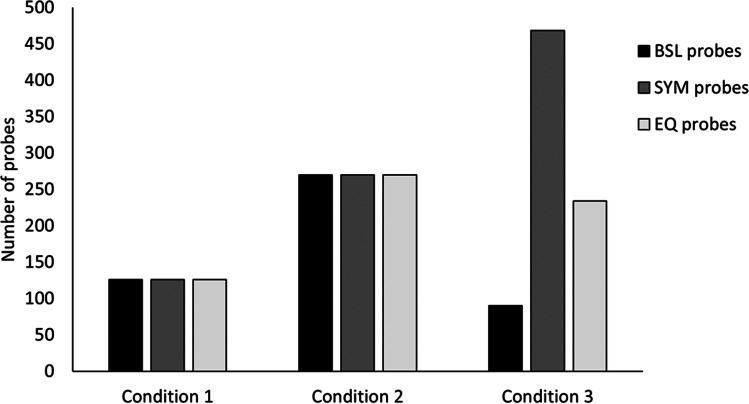
Number of Probes of Each Type Presented per Condition *Note.* BSL = baseline probes; SYM = symmetry probes; EQ = equivalence probes

Differences between the number of correct and incorrect responses during probe trials between conditions could be explained by the static nature of the SIM testing protocol. Using this protocol, participants of Conditions 1 and 2 have been exposed to the same number of probes (BSL, SYM, and EQ; 18 trials each per testing phase). However, the simple-to-complex testing protocol yields a different number of probes because they were presented according to the performance of each individual (see Fig. 5).

Moreover, Fig. 3 shows that most participants who finished the procedure and passed the equivalence test (8/11) tended to learn the second set of baseline relations in fewer trials or with as many trials as the first set. On the other hand, most participants who failed the test or withdrew (4/7) tended to require more trials to learn the second set of baseline relations. These results suggest that the difficulty in acquiring the baseline may have been the cause of failure and dropout. Conditions 1 and 2 generated the same number of withdrawals and/or failures. Therefore, the training structure does not seem to have been a factor in the negative results. Condition 3 generated fewer withdrawals and/or failures. Therefore, the results indicate that the SIM protocol may be a factor for failure in the test and withdrawal.

### Response Speed

**Fig. 6:**
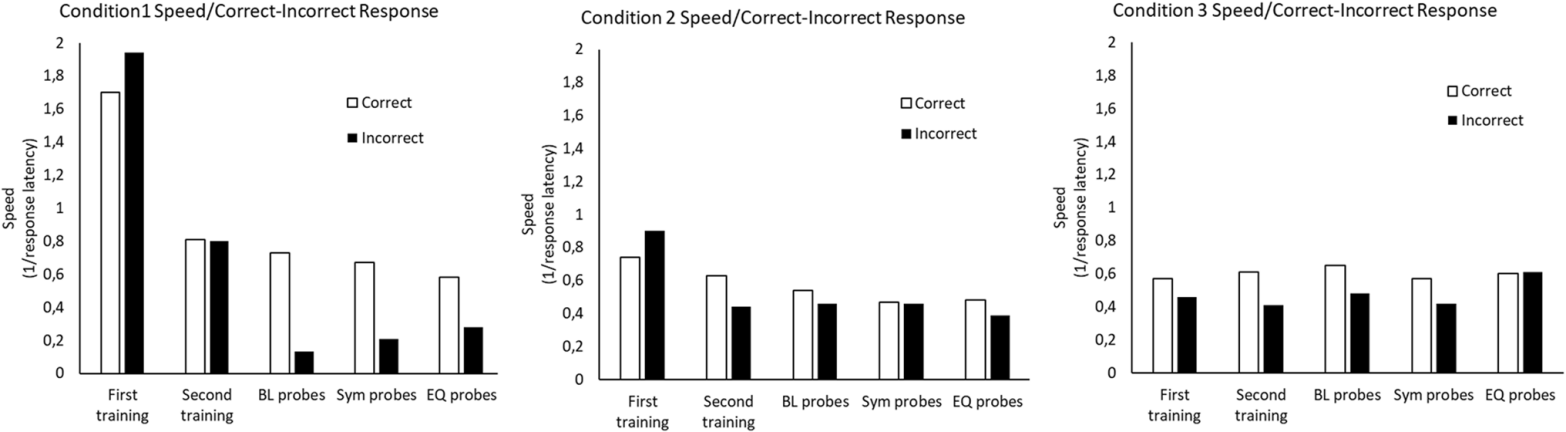
Response Speed Data for Correct and Incorrect Responses in Each Experimental Phase. *Note.* BL = baseline probes; SYM = symmetry probes; EQ = equivalence probes

In general, there is no difference in response speed between conditions. However, we found a faster response in the correct responses (see Fig. 6). Although the response speed mean during the first trained baseline for Condition 1 (OTM-SIM) is superior to the other conditions, this difference could be explained by participant P1004 performance during that phase. P1004 response speed mean was 3.4 s and the mean of the rest of the participants was 0.61 (*SD* = 0.175; see supplementary material for detailed plots of individual performances).

### Participant Variables

**Fig. 7:**
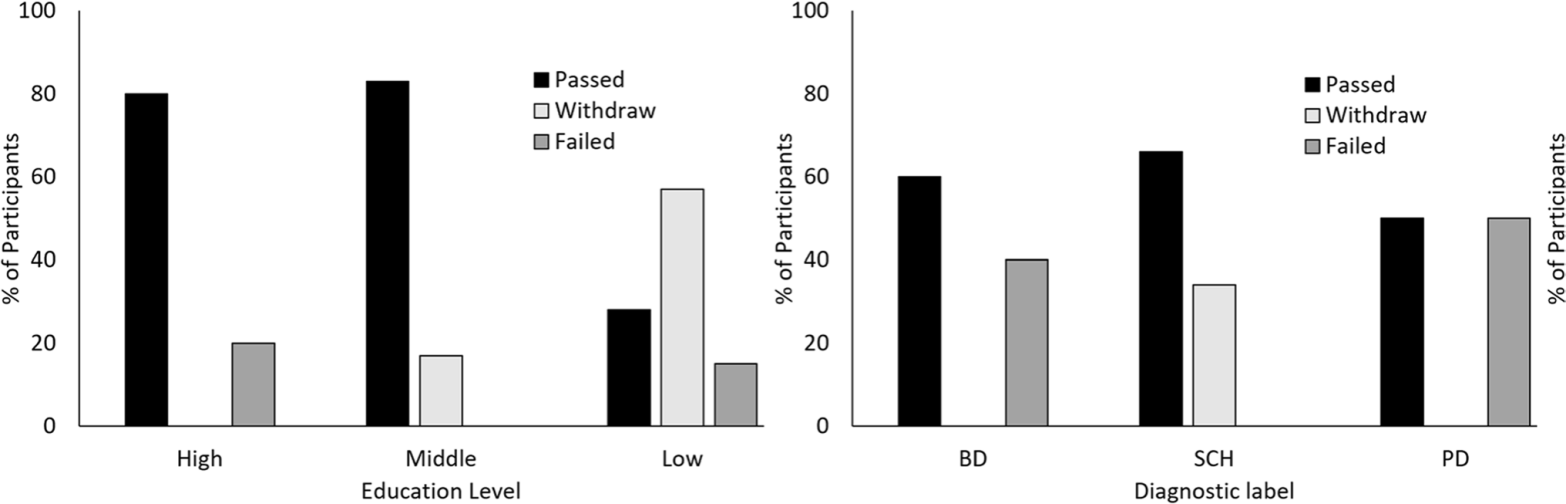
Participants’ Equivalence Formation Performance and Individual Variables. *Note.* Left = performance per education level; Right = performance per diagnostic label; Passed = participants who have met the mastery criteria required for the equivalence class formation; Failed = participants who did not form equivalence classes during the three experimental cycles; BD = bipolar disorder; SCH = Schizophrenia and related psychosis; PD = Personality Disorder

Figure 7 displays the performance of our participants regarding two participant variables that have not been manipulated during the study. High and middle educational levels showed better performance than low educational level participants. Those participants showed more percentage of withdrawal than the other educational level. Finally, we have found no differences in the performance of our participants regarding diagnostic labels.

## Discussion

This study aimed to analyze the stimulus equivalence class formation performance in adults with severe behavioral problems. We wanted to know if this population could perform according to stimulus equivalence with more strict mastery criteria and increase the number of participants in comparison with previous research (e.g., González Pando et al., 2016). Also, we wanted to explore the effects of different training structures and testing protocols in this clinical population.

### General Performance

This experiment adds more evidence about the capability of people diagnosed with severe mental disorders (e.g., schizophrenia, bipolar disorder, and borderline personality disorder) to behave according with stimulus equivalence: 11 out of 13 participants who finished the experiment formed stimulus equivalence classes. In other words, in general participants responded successfully to untrained conditions based on what they had learned during the baseline training. These results are congruent with previous research in stimulus equivalence (e.g., González Pando et al., 2016) and research in derived relations (e.g., O’Neill & Weil, 2014). The work by O’Neill and Weil entails similarities to the protocols used to establish conditional discrimination and the formation of equivalence classes. They wanted to generate deictic relational responding in people diagnosed with schizophrenia. After a baseline training of I–You, Here–There, and Now–Then (as in Weil et al., 2011), all participants (i.e., three adults diagnosed with schizophrenia) increased their performance during trained deictic relational responding, and all participants showed generalization of these responses in novel perspective-taking tasks.

Also, as in previous experiments, we found a high rate of experimental withdrawal; 5 out of 18 participants decided to leave the experiment before finishing the experimental tasks. We found higher withdrawal rates among participants with a lower level of education, and there was no distinction between different diagnostic labels. This could mean that higher levels of education could establish a learning history that could improve the performance during this type of task. However, this conclusion needs to be addressed in further experimental studies.

### OTM and MTO Conditions (Condition 1 vs. Condition 2)

Experimental Conditions 1 and 2 were trained using different training structures (one-to-many and many-to-one, respectively). There is little difference between the performance yielded from both conditions (as in Arntzen & Hansen, 2011; Smeets & Barnes-Holmes, 2005); three participants out of six have responded according to stimulus equivalence in both conditions. OTM condition has indeed yielded a higher rate of participant withdrawal. Still, this difference is not conclusive given the low number of participants in each condition. We can not claim that the OTM training structure yields more withdrawal than MTO in this clinical population. The same is true for the number of trials required to learn baseline relations and the response speed results. The main differences were found in the behavior of just one participant (i.e., P1004); therefore, we cannot claim any essential difference between these two conditions.

### SIM and STC Conditions (Condition 2 vs. Condition 3)

Experimental Conditions 2 and 3 have involved the use of SIM and STC testing protocols, respectively. It is important to note that both conditions were trained with the same MTO structure. STC testing protocol yielded more responses according to stimulus equivalence than the SIM protocol. Also, participants in the STC condition needed fewer trials to learn baseline relations. Also, the STC testing protocol produced fewer incorrect responses than the SIM protocol. This could explain differences in the withdrawal/failure rates between conditions. SCT training protocol (Condition 3) yielded less withdrawal/failure rate than the other conditions with SIM testing protocol (Conditions 1 and 2). These results are in line with previous research found in other populations (e.g., Fienup et al., 2015) and suggest that effective teaching involves the mastery of simple prerequisite behaviors before developing larger repertoires (Skinner, 1968).

### Implications for Applied Research

The present study contributes to the knowledge about individuals diagnosed with severe mental disorders. This study replicates previous research and confirms that people diagnosed with these types of problems can establish novel stimulus equivalence. Also, this study may have opened the door to the use of EBI with people with severe behavioral disorders by providing new experimental data of the training structures and protocols in this population. Although more research is needed to confirm that the STC testing protocol is more efficient than the SIM protocol, the STC testing protocol could be of use when working with this clinical population. These results align with previous research that has indicated that STC protocol stabilized individual differences, yielded better results, and was more efficient (e.g., Fienup et al., 2015). In addition, this paradigm of research can be useful to measure this clinical population's cognitive functioning (e.g., remembering tasks using experimentally established classes). It could be interesting analyzing how this performance varies with, for example, more training or under novel circumstances (e.g., under new pharmacological treatment). Finally, we would like to highlight that we did not find any difference between severe mental disorders’ diagnostics: stimulus equivalence performance was a function of procedure variables (i.e., testing protocols), and previous individual learning history (i.e., educational level). This emphasizes the future significance of an idiographic approach to the assessment and treatment of this clinical population.

### Limitations and Further Research

This study would be strengthened using a different sampling method. Here we included all participants who wanted to participate from a labor rehabilitation center. Future research could use more elaborated sampling methods to control possible biased results. Another limitation of this study was the lack of experimental control of individuals’ pharmacological treatment; they all continued their treatment as usual. This lack of experimental control prevents us from knowing more about pharmacological treatments' interaction with stimulus equivalence phenomena. Future studies could be conducted to measure this potential interaction. Another limitation is the lack of additional measurements of potential motivational variables that could affect the performance of our participants. Gathering more information about the participants’ status would be useful for further research. For instance, our results could be interpreted as an empirical demonstration that this population did not show any deficits in verbal fluency and symbol coding, which could affect equivalence class formation. We encourage to avoid such interpretations because we did not have control and we didn’t have any additional measure. Thus, further studies could increase the number of participants, expand the analysis of stimulus equivalence behavioral phenomena with more classes and members, perform longitudinal studies to analyze this behavior unfolding in time, control the educational level of participants, add aditional measures and analyze the usefulness EBI to teach this clinical population new skills.

## Summary

We have analyzed the stimulus equivalence performance of 18 participants diagnosed with severe mental disorders (e.g., schizophrenia, bipolar disorder, borderline personality disorder, and major depression). Responses according to stimulus equivalence were found in 11 out of 13 participants who had finished the study (5 participants decided to leave before completing the tasks). STC testing protocol yielded better results than the SIM protocol. No differences were found between training structures. Further research focused on the differential effect of these testing protocols could expand the knowledge about stimulus equivalence research in this clinical population. Also, novel studies could study the effect of pharmacological treatment on stimulus equivalence performance and develop EBI in adults with severe behavioral problems.

## Data Availability

The datasets generated during and/or analyzed during the current study are not publicly available due to the clinical data privacy policy but are available from the corresponding author upon reasonable request.

## Footnotes

1 The term *severe behavioral problem* does not imply the use of mental constructs or any underlying biological disorder. As a result, when referring to the problem, we use it as an appropriate substitute for terms like *severe mental disorder* or *serious mental illness*. In addition, we use the term *severe mental disorder* to refer to the diagnostic label given to our participants.
